# Explaining the Microtubule Energy Balance: Contributions Due to Dipole Moments, Charges, van der Waals and Solvation Energy

**DOI:** 10.3390/ijms18102042

**Published:** 2017-09-22

**Authors:** Ahmed Taha Ayoub, Michael Staelens, Alessio Prunotto, Marco A. Deriu, Andrea Danani, Mariusz Klobukowski, Jack Adam Tuszynski

**Affiliations:** 1 Medicinal Chemistry Department, Heliopolis University, Cairo-Belbeis Desert Rd, El-Nahda, El-Salam, Cairo Governorate 11777, Egypt; atayoub@ualberta.ca; 2 Department of Physics, University of Alberta, Edmonton, AB T6G 2E1, Canada; staelens@ualberta.ca; 3 Istituto Dalle Molle di Studi sull’Intelligenza Artificiale (IDSIA), Scuola Universitaria Professionale Della Svizzera Italiana (SUPSI), Università Della Svizzera Italiana (USI), Centro Galleria 2, Manno CH-6928, Switzerland; alessio.prunotto@gmail.com (A.P.); deriu.marco@gmail.com (M.A.D.); andrea.danani@supsi.ch (A.D.); 4 Department of Chemistry, University of Alberta, Edmonton, AB T6G 2G2, Canada; mariusz.klobukowski@ualberta.ca

**Keywords:** microtubules, dipole moment, dynamic instability

## Abstract

Microtubules are the main components of mitotic spindles, and are the pillars of the cellular cytoskeleton. They perform most of their cellular functions by virtue of their unique dynamic instability processes which alternate between polymerization and depolymerization phases. This in turn is driven by a precise balance between attraction and repulsion forces between the constituents of microtubules (MTs)—tubulin dimers. Therefore, it is critically important to know what contributions result in a balance of the interaction energy among tubulin dimers that make up microtubules and what interactions may tip this balance toward or away from a stable polymerized state of tubulin. In this paper, we calculate the dipole–dipole interaction energy between tubulin dimers in a microtubule as part of the various contributions to the energy balance. We also compare the remaining contributions to the interaction energies between tubulin dimers and establish a balance between stabilizing and destabilizing components, including the van der Waals, electrostatic, and solvent-accessible surface area energies. The energy balance shows that the GTP-capped tip of the seam at the plus end of microtubules is stabilized only by −9 kcal/mol, which can be completely reversed by the hydrolysis of a single GTP molecule, which releases +14 kcal/mol and destabilizes the seam by an excess of +5 kcal/mol. This triggers the breakdown of microtubules and initiates a disassembly phase which is aptly called a catastrophe.

One of the most interesting features of microtubules (MTs) is their dynamic instability. In fact, most of the functions that MTs perform inside the cell are due to their ability to undergo fast transitions between growing and shrinking phases [1]. The issue of microtubule stability has been extensively studied, but is still incompletely understood, since a generally accepted mechanistic explanation of what causes these drastic changes in microtubule stability including a quantitative energy balance analysis is missing. Several models have been developed in recent years, trying to explain how microtubule dynamics is regulated; i.e., the parameters causing growth or shrinkage, and how cells are able to modify such parameters according to their needs and means. However, an unequivocal, well-established model still has to be built. Indeed, the variables that need to be taken into account are numerous, and a combination of all of these elements is usually difficult to achieve.

Several studies have been performed which investigated major aspects of the binding effects between tubulin dimers in microtubules, but no study has yet been produced that would analyze these effects in their totality. Sept et al. studied the solvation effects in 2003, and showed that the B-lattice microtubule structure was slightly more stable than its A-lattice counterpart [2], which agrees with the propensity of tubulin ensembles in vitro and in vivo to polymerize into B-lattice structures. Similarly, Drabik et al. calculated the potential of mean force between tubulin protofilaments and arrived at the same conclusion, but generalized this analysis to various tubulin isotypes [3]. In 1981, Erickson and Pantaloni calculated the entropic contributions to the total energy profile using a theory based on a model of rigid subunits and bonds and simple principles of thermodynamics [4]. They estimated a critical supersaturation ratio of subunit concentrations of 3.5 to 7, which is comparable to the experimental value of 1.5 to 3. We previously calculated the contribution of hydrogen bonds to the lateral and longitudinal tubulin binding energies using density functional theory [5], showing that they represent a major factor contributing to MT stability. We also identified specific residues responsible for hydrogen bond formation. We also found that lateral contacts are stronger than longitudinal ones. We then followed it by a complete microtubule simulation, resulting in a molecular mechanics/generalized Born surface area (MMGBSA) estimation of the tubulin binding energies [6]. This study also showed that the microtubule seam is the most energetically labile inter-dimer interface, and therefore could provide a trigger for MT disassembly. In this study, we applied molecular mechanics force field equations to estimate the electrostatic and van der Waals interaction terms. On the other hand, we employed the generalized Born model to estimate the polar solvation term and the surface area method to estimate the non-polar solvation term [7]. In the present paper, we intend to quantitatively estimate energetic contributions to MT stability, giving a break-down according to the type of physical interaction involved: charge–charge, dipole–dipole, van der Waals, and solvent-accessible surface area interactions.

In the present work, we have made an estimate of the energy balance for a microtubule structure that includes all dimer–dimer interaction contributions to microtubule stability, most of which have been individually estimated in earlier publications. However, a missing energy component in the previous studies was the dipole–dipole interaction energy, which involved not only the nearest neighbours of a dimer in contact with it but also all remaining dipole moments in a microtubule. Because the dipole–dipole interaction is mainly through protein and not through water, one must take into account its long-range character, as ionic screening is not effective in this case. Moreover, the dielectric constant for the protein is much lower than that for water, making this interaction stronger than a comparable dimer–dimer interaction in solution. We have calculated this component according to the formula:

(1) $$U_{int}=\frac{1}{4\pi \varepsilon \varepsilon_{0}r^{3}}\left(p_{1}\cdot p_{2}-3\left(p_{1}\cdot \hat{r}\right)\left(p_{2}\cdot \hat{r}\right)\right)$$

where $p_{1}$ and $p_{2}$ are the dipole moments of the dimers, *r* is the distance between them, and $\hat{r}$ is the unit vector pointing from one dimer to the other. $\varepsilon_{0}$ is the permeability of free space, and ε is the dielectric constant of the protein. The calculation is described in detail the Supplementary Material utilizing dipole moment values calculated previously for tubulin elsewhere [8,9]. As a result of this estimate, a total value of repulsive dipole–dipole interaction of 27 kcal/mol was obtained. In Table 1, a balance of different energetic components that contribute to microtubule stability before disassembly (GTP cap) and after disassembly (GDP cap), is presented. It should be noted that the electrostatic component in MMGBSA calculations does not include the dipole–dipole interaction term, and therefore there is no double counting.

Here we define disassembly as the first step of breakdown, where lateral bonds along the seam are broken at the tip of the plus end of microtubules (see Figure 1). Electrostatic interaction energy, van der Waals (vdW) interaction energy, and interaction energy due to solvent-accessible surface area (SASA) are all obtained from our previous work [6], where details of these atomic-level calculations are given. The values represent interactions at the tip of the seam of microtubules only, since it is the trigger for microtubule disassembly [6,10]. The dipole–dipole interaction energy was calculated for a tubulin dimer at the seam (the highlighted dimer in Figure 1), and its interactions with all dimers in a microtubule are accounted for. The value remains essentially the same at 27 kcal/mol before and after disassembly since, according to our definition of disassembly, the orientation of dimers does not change significantly at the start of the disassembly process. The same is true for electrostatic interactions. However, SASA and vdW interactions break down completely at the start of disassembly since they are short-ranged involving contacts with a neighbouring dimer. Electrostatics, vdW, and SASA values listed in the table represent lateral interactions at the seam interface only, because this is the one that breaks down first (Figure 1).

As Table 1 shows, the total value of all energetic contributions before disassembly is −9 kcal/mol. This negative free energy favors a stable microtubule structure as long as the GTP cap is present. As soon as the single GTP molecule that is bound to the beta tubulin unit in the dimer at the seam (highlighted in Figure 1) hydrolyzes, it releases a free energy of nearly +14 kcal/mol [11]. This free energy offsets the negative binding free energy of the stable state of the microtubule by 5 kcal/mol, tipping the balance to an energetically unstable state, which triggers microtubule disassembly in a domino-like fashion. This is because the dimers forming the cylindrical MT structure below the so-called lateral GTP cap have their beta units with GDP molecules bound to them according to the lateral cap hypothesis. Therefore, disassembly starts by the breakdown of the lateral bonds at the seam, one by one, where it starts to unfold as shown in Figure 1. This leads to the complete breakdown of all the short-range bonds, including vdW and SASA bonds. As a result, the sum total of the interaction energies after the start of disassembly approaches +119 kcal/mol (see Table 1), which drives even more disassembly and repulsion between neighbouring lateral dimers. Since the tip of the microtubule is what provides the lattice constraints which keep the entire microtubule together, this breakdown at the seam would initiate a total break down of the entire structure, and other lateral contacts around the cylinder would break following a free energy pathway similar to the one at the seam.

In summary, in this paper we have been able to quantitatively explain the origin of the dynamic instability of microtubules by providing a detailed energy balance before and after GTP hydrolysis at the tip involving a tubulin dimer located at the seam. Further work on differences between tubulin isotypes and tubulin from various species [12] as well as mutants can now be undertaken using the blueprint provided in this paper by recalculating the contributions from individual physical interactions between the dimers. This could shed light not only on the differences in their stabilities, but also the different dynamic properties which are due to a change in the respective energy balances. The latter property has been known for over two decades [13], but has never been mechanistically explained.

In order to discuss the implications of the MT lattice energetics on the MT dynamics, it should be stressed again that the lateral contacts are weaker than the longitudinal ones. Moreover, the lateral contacts at the seam are still weaker than the other lateral contacts, which is evident in the literature [2,6]. While this does not necessarily indicate that the seam is the trigger for microtubule catastrophe, its conformational state may affect the rest of the lattice via conformational and dynamical interactions. Experimental observations indeed correlate the size of the stabilizing cap [14,15] and the emergence of destabilizing plus-end structures [16,17,18] with the probability of catastrophes. However, there may be additional subtle considerations to include in this analysis. For example, the MT lattice vibrates under thermal motion, and in particular [19], the most prevalent form of vibrational motion is “bending”. Bending is determined mostly by adjacent protofilaments’ shear stresses. If the lateral interaction is weaker at the seam, this may cause a discontinuity in the shear distribution much more readily in the B than in the A lattice. In our opinion, this effect might cause the bending to proceed along a certain direction or destabilize the seam in a torsional manner (again via lateral interactions). As in any condensed state material, the stress propagates quickly in the form of structural defects. Hence, we do not necessarily see it as affecting only a single tubulin layer, but possibly several—especially those already weakened by GTP hydrolysis events which may occur spontaneously at isolated locations throughout the structure of the MT lattice. This type of behaviour is stochastic, and can be quite consistent with various experimental data [14,15,20,21]. In this dynamical process of structural weakening and eventual collapse, the cap certainly plays a role in maintaining the lattice stable, but the molecular reason for the stability break may also depend on this lateral interaction difference between seam and the rest of the wall. A proper implementation of this type of reasoning into a mechanistic mathematical model lies outside the scope of this paper, but the detailed energetics of the MT lattice this paper summarized will be of use in building such a model.

## Figures and Tables

**Figure 1:**
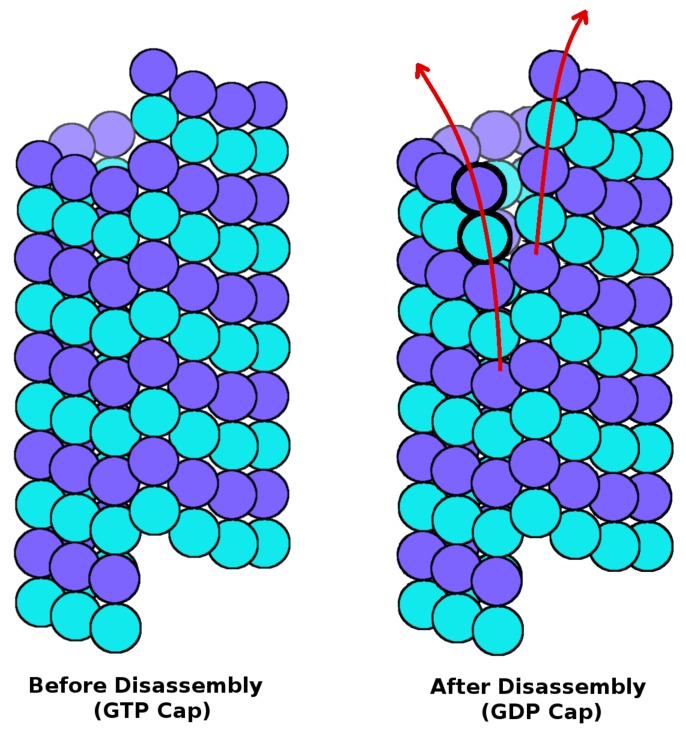
A schematic diagram of microtubule (**left**) before disassembly, where a GTP cap is present, and (**right**) right after the start of disassembly, after GDP hydrolysis and breakdown of lateral contacts at the seam. The plus end is at the top and minus end is at the bottom. β-tubulin is coloured purple and α-tubulin is colored cyan. Red arrows represent the direction of outward curling during disassmbly.

**Table 1:** Energy balance of different components of interaction energies between tubulin dimers at the tip of the seam of a microtubule (in kcal/mol) before and after disassembly.

Component	Before Disassembly	After Disassembly
Dipole–Dipole	27	27
vdW	−101	0
Electrostatic + GB	84	92
SASA	−19	0
Total	−9	119

Electrostatic interactions are represented as a sum of charge–charge interactions plus solvent screening calculated through the generalized Born (GB) method. SASA: solvent-accessible surface area; vdW: van der Waals.

## Supplementary Materials

Supplementary Material can be found at www.mdpi.com/1422-0067/18/10/2042/s1.
