# Supplementary material for: Explaining the Microtubule Energy Balance: Contributions Due to Dipole Moments, Charges, van der Waals and Solvation Energy
Source: Int J Mol Sci. 2017 Sep 22;18(10):2042. doi: 10.3390/ijms18102042 (PMC5666724; doi:10.3390/ijms18102042)
Supplement: Supplementary file 1 [file ijms-18-02042-s001.pdf]

Brief Report

Explaining the Microtubule Energy Balance: Contributions Due to Dipole Moments, Charges, van der Waals and Solvation Energy

Ahmed Taha Ayoub [1], Michael Staelens [2], Alessio Prunotto [3], Marco A. Deriu [3], Andrea Danani [3], Mariusz Klobukowski [4] and Jack Adam Tuszynski [2,*]

1 Medicinal Chemistry Department, Heliopolis University, Cairo-Belbeis Desert Rd, El-Nahda, El-Salam , Cairo Governorate 11777, Egypt; atayoub@ualberta.ca
2 Department of Physics, University of Alberta, Edmonton, AB T6G 2E1, Canada; staelens@ualberta.ca
3 Istituto Dalle Molle di Studi sull'Intelligenza Artificiale (IDSIA), Scuola Universitaria Professionale Della Svizzera Italiana (SUPSI), Università Della Svizzera Italiana (USI), Centro Galleria 2, Manno CH-6928, Switzerland; alessio.prunotto@gmail.com (A.P.); deriu.marco@gmail.com (M.A.D.); andrea.danani@supsi.ch (A.D.)
4 Department of Chemistry, University of Alberta, Edmonton, AB T6G 2G2, Canada; mariusz.klobukowski@ualberta.ca
* Correspondence: jackt@ualberta.ca; Tel.: +1-780-432-8906

Received: 14 August 2017; Accepted: 13 September 2017; Published: 22 September 2017

1. Methods of Calculation of Tubulin Dipole Moment Interactions

Dipole moment contributions of each tubulin dimer are shown as arrows in Figure S1.

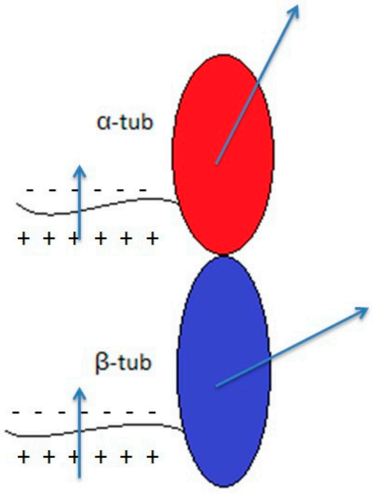

Figure S1. This is the configuration which shows how the electric dipole moments that we will use are disposed. There are two contributions that are associated with each monomer, one related to the monomer body and the other one related to the C-terminal tail.

Considering one tubulin dimer, the electric dipole moments that are considered to participate in a non-negligible way are the ones that are associated with the bodies of the two tubulin monomers, α and β, and the ones associated with the two C-terminal tails. Due to the absence of a crystal structure, we adopted a stretched configuration for the C-terminal tails. This is probably the most statistically significant configuration. In this configuration, the counterions concentrate on the top of the C-terminal tail, generating an electric dipole moment whose only non-zero component is the vertical one (see Figure S2).

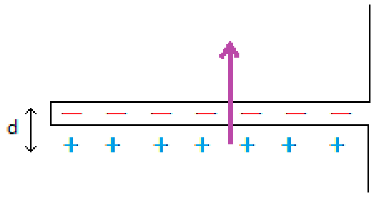

Figure S2. A representation of the model that was used to calculate the electric dipole moment for the C-terminal tails. Charges are uniformly distributed along the length of the C-terminal tail and each of them is neutralized by a positive counterion that is placed into the medium opposite to a negative charge, at a distance (*d* in the figure) that is approximately equal to 1 nm in water. The violet arrow represents the electric dipole moment.

The center of masses of tubulin dimers in a 78-dimer microtubule were obtained from a previous model [1]. The model thus contained 6 layers of microtubule rings, each having 13 tubulin dimers. We were interested in calculating the dipole-dipole interaction energy between the highlighted dimer in Figure 1 in the main text and all of the remaining dimers in the system. In order to calculate interaction energy between any two tubulin dimers, the following formula was used,

$$U_{int} = \frac{1}{4\pi\varepsilon\varepsilon_0 r^3}\left(p_1 \cdot p_2 - 3(p_1 \cdot \hat{r})(p_2 \cdot \hat{r})\right) \quad (1)$$

where p_1 and p_2 are the dipole moments of the dimers, r is the distance between them and $\hat{r}$ is the unit vector pointing from one dimer to the other. $\varepsilon 0$ is the permeability of free space and ε is the dielectric constant of the protein which is equal to 8.4 for tubulin [2]. A MATLAB script was written to calculate the interaction energy between the dimer of interest and all the remaining 77 dimers in the system.

For each tubulin dimer, four dipole moment values were summed up into an average dipole moment that includes; dipole moment of α-tubulin body, β-tubulin body, α-tubulin C-terminal tail and β-tubulin C-terminal tail. The dipole moment values for the bodies were obtained from [3,4]. The dipole moment values for the tails were obtained using the information about the charge carried by each C-terminal tail, and assuming that the charges are uniformly linearly disposed along the C-terminal tail, and that for each charge, a counterion is present that neutralizes it (Figure S1). The counterions are considered to be at a distance that is equal to the Bjerrum length, that is about 1 nm for what concerns a physiological solution. We made sure that the dipole moment vector is rotated about the z axis by (4π/13) using rotation matrices as we go around the microtubule cylinder. All of the tubulin sequences used in the calculations were porcine tubulin.

References

1. Ayoub, A.T.; Klobukowski, M.; Tuszynski, J.A. Detailed Per-residue Energetic Analysis Explains the Driving Force for Microtubule Disassembly. *PLoS Comput. Biol.* **2015**, *11*, 1–21.
2. Mershin, A.; Kolomenski, A.; Schuessler, H.; Nanopoulos, D. Tubulin dipole moment, dielectric constant and quantum behavior: computer simulations, experimental results and suggestions. *Biosystems* **2004**, *77*, 73 – 85.
3. Tuszynski, J.A.; Carpenter, E.J.; Huzil, J.T.; Malinski, W.; Luchko, T.; Luduena, R.F. The Evolution of the Structure of Tubulin and its Potential Consequences for the Role and Function of Microtubules in Cells and Embryos. *Int. J. Dev. Biol.* **2006**, *50*, 341–58.
4. Carpenter, E.J.; Huzil, J.T.; Ludueña, R.F.; Tuszynski, J.A. Homology Modeling of Tubulin: Influence Predictions for Microtubule's Biophysical Properties. *Eur. Biophys. J.* **2006**, *36*, 35–43.
